# Catalytic activity and stereoselectivity of engineered phosphotriesterases towards structurally different nerve agents in vitro

**DOI:** 10.1007/s00204-021-03094-0

**Published:** 2021-06-23

**Authors:** Anja Köhler, Benjamin Escher, Laura Job, Marianne Koller, Horst Thiermann, Arne Skerra, Franz Worek

**Affiliations:** 1 Institut für Pharmakologie und Toxikologie der Bundeswehr, 80937 Munich, Germany; 2 Lehrstuhl für Biologische Chemie, Technische Universität München, 85354 Freising, Germany

**Keywords:** Bispecific bioscavenger, Detoxification, Mutant, Nerve agent, PASylation, Phosphotriesterase

## Abstract

Highly toxic organophosphorus nerve agents, especially the extremely stable and persistent V-type agents such as VX, still pose a threat to the human population and require effective medical countermeasures. Engineered mutants of the *Brevundimonas diminuta* phosphotriesterase (BdPTE) exhibit enhanced catalytic activities and have demonstrated detoxification in animal models, however, substrate specificity and fast plasma clearance limit their medical applicability. To allow better assessment of their substrate profiles, we have thoroughly investigated the catalytic efficacies of five BdPTE mutants with 17 different nerve agents using an AChE inhibition assay. In addition, we studied one BdPTE version that was fused with structurally disordered PAS polypeptides to enable delayed plasma clearance and one bispecific BdPTE with broadened substrate spectrum composed of two functionally distinct subunits connected by a PAS linker. Measured *k*_cat_/*K*_M_ values were as high as 6.5 and 1.5 × 10^8^ M^−1^ min^−1^ with G- and V-agents, respectively. Furthermore, the stereoselective degradation of VX enantiomers by the PASylated BdPTE-4 and the bispecific BdPTE-7 were investigated by chiral LC–MS/MS, resulting in a several fold faster hydrolysis of the more toxic P(−) VX stereoisomer compared to P(+) VX. In conclusion, the newly developed enzymes BdPTE-4 and BdPTE-7 have shown high catalytic efficacy towards structurally different nerve agents and stereoselectivity towards the toxic P(−) VX enantiomer in vitro and offer promise for use as bioscavengers in vivo.

## Introduction

Organophosphorus (OP) nerve agents are extremely hazardous compounds that were repeatedly used in military conflicts and by terrorists. Recent nerve agent attacks with sarin in Syria since 2013, assassinations with VX in Malaysia 2017 and with Novichok agents in UK 2018 and Russia 2020 underline the ongoing threat and the necessity for effective medical countermeasures (Costanzi et al. 2018; John et al. 2018; Steindl et al. 2021).

The acute toxicity of nerve agents is based on the covalent binding to a serine residue at the active site of acetylcholinesterase (AChE) that leads to inhibition of AChE activity and inadequate breakdown of the neurotransmitter acetylcholine (ACh) in the synaptic clefts of the peripheral and central nervous system. In consequence, muscarinic and nicotinic receptors become overstimulated, resulting in severe signs of cholinergic crisis, which may ultimately lead to death by suffocation (Grob 1963; Holmstedt 1959; Worek et al. 2016b).

OP nerve agents can be divided into two major groups: the volatile G-agents, which include tabun (GA), sarin (GB), soman (GD), and cyclosarin (GF), and the highly persistent and less volatile V-agents such as VX, Russian VX (VR) and Chinese VX (CVX) (Rice 2016). A common characteristic of nerve agents is a chiral center at the phosphorus atom that leads to P(+) and P(−) stereoisomers, whereas soman has a second chiral center in a side chain, leading to four diastereomers (Benschop and de Jong 1988). These stereoisomers exhibit different biological properties such as a higher biological stability and a higher inhibitory potency towards human AChE for the P(−) isomers (Benschop and de Jong 1988; Tenberken et al. 2010a). In case of VR(−), the inhibitory potency is 22,000-fold higher than that of VR(+) and with VX the ratio VX(−)/VX(+) is 380 (Tenberken et al. 2010b; Reiter et al. 2015).

Standard treatment of OP poisoning consists of rapid administration of a muscarinic antagonist, usually atropine, and an oxime (e.g., obidoxime) to reactivate inhibited AChE. This basic therapy may be supplemented by an anticonvulsant drug for neuroprotection and termination of seizures (Thiermann et al. 2016; Marrs and Sellström 2007). Unfortunately, oxime efficacy is limited by narrow OP substrate specificity and inadequate blood–brain-barrier penetration, which results in insufficient treatment of poisonings by different nerve agents (Worek et al. 2020).

A potentially promising therapeutic approach is the use of catalytic bioscavengers that can detoxify various OPs via enzymatic hydrolysis (Nachon et al. 2013). During the last decades, mutants of the naturally occurring phosphotriesterase from *Brevundimonas *(*Pseudomonas*)* diminuta* (BdPTE) were generated to serve as catalytic bioscavengers against OP nerve agents. The metal-dependent hydrolase is a homodimer of 329 amino acids per subunit. The active site is formed by a pair of zinc ions that are bound by several His side chains and a carbamylated Lys side chain and are bridged by a nucleophilic hydroxide ion. OP hydrolysis is induced by the nucleophilic attack of this hydroxide at the phosphorus center, whereby in case of OP nerve agents a preferential degradation of P(+) enantiomers by the wild-type BdPTE was shown (Tsai et al. 2010; Cherny et al. 2013; Goldsmith et al. 2017).

Several research groups have optimized native BdPTE with regard to expression yield, stability (Roodveldt and Tawfik 2005) as well as substrate specificity, most importantly by inverting the stereopreference to the more toxic P(−) enantiomer (Chen-Goodspeed et al. 2001b, 2001a; Bigley et al. 2013, 2015; Goldsmith et al. 2016; Tsai et al. 2010, 2012). Subsequent optimization of the BdPTE mutant C23 (Cherny et al. 2013) by Goldsmith et al. (2017) resulted in the variants 10-2-C3 and 10–2-C3(I106A) which exhibit even higher catalytic activity towards V-agents. Furthermore, Job et al. (2020) increased the oxidation stability of BdPTE 10-2-C3 by replacing two free Cys to Val, resulting in the variant 10-2-C3(C59V/C227V). Recent investigations by Escher et al. (2020) led to an enlarged substrate spectrum by creating bispecific enzymes, e.g., by fusing two different PTE monomers via a 100-residue Pro/Ala/Ser (PAS) linker (Schlapschy et al. 2013) to obtain a heterodimeric single-chain PTE (scPTE).

Notably, PTEs with catalytic efficiencies ≥ 10^7^ M^−1^ min^−1^ towards the more toxic P(−) nerve agent enantiomers should enable a rapid break down in vivo at low bioscavenger doses (< 1 mg kg^−1^); however, the BdPTE enzymes suffer from a short circulation half-life of merely ~ 1 h in rodents (Despotović et al. 2019; Worek et al. 2016a; Ashani et al. 2016). Therefore, BdPTE mutants with markedly improved biological stability are needed for a successful post-exposure therapy, especially in case of percutaneous poisoning by V-type nerve agents with prolonged persistence (Worek et al. 2014, 2016a). A proven strategy to delay clearance of pharmacologically active proteins in vivo is the PASylation technology (Binder and Skerra 2017). Depending on the length of the conformationally disordered and biochemically inert polypeptide chain comprising the amino acids Pro, Ala, and Ser the hydrodynamic volume of the protein drug increases dramatically, thus delaying kidney filtration (Schlapschy et al. 2013).

In the present study, we have characterized the catalytic activities of diverse BdPTE mutants as well as the PASylated BdPTE-4 and the newly engineered scPTE, dubbed BdPTE-7, towards a spectrum of four G-  and 10 V-type nerve agents as well as three amiton OP analogues. Moreover, the stereoselective degradation of VX by the PASylated BdPTE candidates was analyzed by chiral LC–MS/MS.

## Materials and methods

### Chemicals

17 V- and G-type nerve agents as well as amiton analogues were made available by the German Ministry of Defence, prepared as 0.1% v/v stock solution in acetonitrile and stored at ambient temperature. Pursuant to regulations of the Organization for the Prohibition of Chemical Weapons (OPWC), OP nerve agents were handled exclusively within the Bundeswehr Institute of Pharmacology and Toxicology. Tris(hydroxymethyl)-aminomethane (TRIS), acetylthiocholine (ATCh) and 5,5′-dithiobis(2-nitrobenzoic acid) (DTNB) were purchased from Sigma-Aldrich (Taufkirchen, Germany), whereas acetylcholinesterase (AChE) was prepared from human erythrocytes according to Bierwisch et al. (2014) and Dodge et al. (1963). All other chemicals were supplied by Merck (Darmstadt, Germany) or Carl Roth (Karlsruhe, Germany).

### Plasmid construction

The synthetic genes encoding the BdPTE homodimer variants BdPTE-1 and BdPTE-2 (Table 1) were obtained from a previous study (Job et al. 2020) and cloned on the expression plasmid pASK-IBA5(+), also encoding the N-terminal *Strep*-tag II (Schmidt and Skerra 2007). Amino acid substitutions were introduced into BdPTE-2 by QuikChange site-directed mutagenesis (Agilent, Santa Clara, CA) with appropriate oligodeoxynucleotide pairs (Eurofins, Ebersberg, Germany) to yield BdPTE-3, BdPTE-5 and BdPTE-6 (see Table 1). The *Sap*I restriction site in front of the stop-codon of the variant BdPTE-3 was used to insert a PAS#1(200) gene cassette according to Schlapschy et al. (2013), yielding BdPTE-4. The expression plasmid encoding the heterodimeric variant BdPTE-7 composed of BdPTE-1 and BdPTE-6 subunits, fused by a PAS(100) linker between the C-terminus of BdPTE-1 and the N-terminus of BdPTE-6 and complemented by electrostatic steering mutations R152E as well as E71K to stabilize the heterodimerization, was constructed according to Escher et al. (2020). For the additional C-terminal PASylation, two inverted *SapI* restriction sites were inserted upfront of the stop-codon via PCR, followed by the seamless insertion of a PAS#1(200) gene cassette as above.

**Table 1:** Overview of tested BdPTE variants

BdPTE-1	C23^(a)^
BdPTE-2	10-2-C3^(b)^
BdPTE-3	10-2-C3(C59V/C227V)^(c)^
BdPTE-4	10-2-C3(C59V/C227V)-PAS(200)
BdPTE-5	10-2-C3(I106A)^(b)^
BdPTE-6	10-2-C3(I106A/C59V/C227V)^(c)^
BdPTE-7	BdPTE-C23(R152E)-PAS(100)-10-2-C3(I106A/C59V/C227V/E71K)-PAS(200)

Published by (a) Cherny et al. (2013); (b) Goldsmith et al. (2017); (c) Job et al. (2020)

### Protein expression and purification

The BdPTE variants were produced in *E. coli* BL21 and purified as previously described (Escher et al. 2020; Job et al. 2020). Briefly, bacteria were cultivated in shake flasks with 2 L LB medium supplemented with 100 mg L^−1^ ampicillin and 0.2 mM ZnSO_4_ and induced with 200 µg L^−1^ anhydrotetracycline. Bacteria harboring the homo-dimeric BdPTE versions were induced at OD_550_ ≈ 0.6 and incubated at 30 °C for up to 5 h, whereas the PASylated BdPTE variants were cultivated in TB medium at 22 °C and induced at OD_550_ ≈ 1.8–2.2 for up to 15 h. Bacteria were harvested by centrifugation (40 min, 4500 rpm, 4 °C) and the cell pellet was resuspended in 3 mL affinity chromatography buffer (100 mM Tris-HCl, 150 mM NaCl, 10 mM NaHCO_3_, 0.1 mM ZnSO_4_, pH 8.0) per 1 g wet weight and disrupted with a high pressure homogenizer (GEA Niro Soavi, Lübeck, Germany). The soluble cell extract was loaded onto a *Strep*-Tactin column for streptavidin affinity chromatography (SAC) according to Schmidt and Skerra (2007). In case of BdPTE-4, the SAC eluate was dialyzed overnight against 20 mM Bis–Tris/HCl, 20 mM NaCl, 10 µM ZnSO_4_ at pH 6.0 and loaded onto a 6 mL ResQ anion-exchange chromatography (AEX) column (GE Healthcare, Freiburg, Germany), equilibrated with the same buffer used during dialysis. In this case, the enzyme did not bind to the column and was collected in the flow-through. For BdPTE-7, a buffer containing 20 mM Hepes/HCl, 20 mM NaCl, 10 µM ZnSO_4_ at pH 7.0 was used for dialysis as well as AEX. Bound scPTE was eluted by a linear concentration gradient from 20 to 250 mM NaCl in running buffer over 20 column volumes. Subsequently, all BdPTE mutants were subjected to size-exclusion chromatography (SEC), performed on a 120 mL HiLoad Superdex 200 16/60 prep grade column or 320 ml HiLoad Superdex 200 26/60 prep grade column (GE Healthcare) using SEC running buffer (50 mM Tris-HCl, 100 mM NaCl, 10 mM NaHCO_3_, 0.01 mM ZnSO_4_, pH 8.0). NaHCO_3_ was omitted for BdPTE-4 and BdPTE-7. Protein concentration of BdPTE-7 was determined by measuring the absorbance at 280 nm on a NanoDrop 2000 spectrometer (Thermo Scientific, Rockford, IL), whereas the concentrations of all other BdPTE variants were measured using an Ultrospec 2100 pro UV/Vis spectrophotometer (GE Healthcare). Molar absorption coefficients of enzyme variants were calculated according to Wilkins et al. (1999) and Gasteiger et al. (2005). All enzyme variants were obtained with > 95% purity, as confirmed by Coomassie-stained sodium dodecyl sulfate polyacrylamide gel electrophoresis (SDS-PAGE) using the buffer system of Fling and Gregerson (1986).

### Enzyme activity measurement

Detoxification of OP by BdPTE variants was quantified by a human AChE inhibition assay in duplicates as previously described (Job et al. 2020; Goldsmith et al. 2016). 500 µL OP solution was added to 100 µL of the appropriately diluted purified recombinant enzyme in TN-buffer (50 mM Tris–HCl, 50 mM NaCl, pH 8) and incubated at 37 °C. After specific time points (1, 7, 14, 20, 30, 45, 60, 90, and 120 min), 50 µL incubate was transferred to a polystyrol cuvette (PS macro, VWR, Darmstadt, Germany) prefilled with 3 mL phosphate buffer (0.1 M, pH 7.4) and 0.1 mL DTNB (10 mM). Then, 10 µL AChE and 50 µL ATCh (28.4 mM) were added and AChE inhibition curves were recorded for 5 min at 37 °C and 412 nm (UV-2600, Shimadzu, Kyoto, Japan). Inhibition curves were analyzed by non-linear regression analysis to obtain the pseudo first-order rate constant *k*_AChE_(*t*_OPH_). The first-order OP degradation constant of the BdPTE variant *k*_OPH_ was obtained from a plot of *k*_AChE_(*t*_OPH_) *versus* time, and the corresponding second-order rate constant (the catalytic efficiency *k*_cat_/*K*_M_) was calculated using the following equation, wherein [*E*] is the final concentration of BdPTE in the incubate with OP:

1 $$k_{{{\text{cat}}}} /K_{{\text{M}}} = \frac{{k_{{{\text{OPH}}}} }}{{\left[ E \right]}}.$$

### Stereoselective detoxification of VX enantiomers by BdPTE variants

To investigate the stereoselective hydrolysis of VX by BdPTE-4 or BdPTE-7, a mixture of 1250 µL VX and 250 µL diluted enzyme in TN-buffer (pH 8.0; *n* = 3) with final concentrations of 3.3 × 10^–6^ M VX and 2.89 × 10^–9^ M BdPTE-4 or 5.5 × 10^–9^ M BdPTE-7 was incubated at 37 °C. The enzymatic hydrolysis of VX was stopped at defined incubation time points (1, 5, 10, 15, 20, 30, 45, 60, 90, 120, 240, 480, 1440, and 2880 min) by adding 10 µL acetonitrile to 90 µL incubate. Samples were shock frozen in liquid nitrogen and stored at − 80 °C until processing.

For the quantitative analysis of the individual VX enantiomers, samples (100 µL) were diluted in 900 µL deionized water, spiked with 5 ng mL^−1^ VR as internal standard and purified by a preconditioned (1 mL methanol, 1 mL water) SPE cartridge (Strata-X PRP, 30 mg, 1 mL, Phenomenex, Aschaffenburg, Germany) followed by a wash with 1 mL deionized water and elution with 500 µL acetonitrile. Solvent was evaporated from the elutate for 60 min at ambient temperature using a rotating vacuum centrifuge (RVC 2-18 CD plus, Christ, Osterode, Germany) and reconstituted in 100 µL purified water. 10 µl of the solution was injected into an LC–MS/MS system comprising an LC 1260 (binary pump, autosampler, degasser, column oven) and a 6420 Triple-Quad mass spectrometer (both from Agilent Technologies, Waldbronn, Germany). VX enantiomers were base-line separated using a Reprosil-AGP column (150 × 2.0 mm; Altmann Analytics, Munich, Germany) at 30 °C at a flow rate of 175 µL min^−1^ with a gradient of solvent A (25 mM ammonium formate, pH 8.5 in water) and solvent B (25 mM ammonium formate, pH 8.5 in 1:1 methanol–water): 10/10, 20/10–50, 10/50, 1/50–10, 4/10 *t* (min)/*B* (%). The mass fragments as well as fragmentor voltage and collision energies (at a cell accelerator voltage of 7 V) were recorded for the precursor ions of VX *m*/*z* 268.2 → 128.2 (105 V, 13 V), *m*/*z* 268.2 → 79.1 (105 V, 37 V) or VR *m*/*z* 268.2 → 100.2 (110 V, 17 V), *m*/*z* 268.2 → 72.2 (110 V, 29 V) via MS after positive electrospray ionization (ESI) at 1.75 kV using the following conditions: 30 psi ion source gas at a flow of 9 L min^−1^ with a temperature (TEM) of 300 °C and Dwell time of 1000 ms. The LC–MS/MS system was operated using the Agilent MassHunter Workstation Data Acquisition for Triple-Quad and data analysis was performed using Agilent MassHunter Qualitative Analysis software.

### Data analysis

Data analysis, calculations and statistical comparisons were performed using GraphPad Prism Version 5.04 (GraphPad Software, San Diego, CA). Generally, data are presented as mean ± standard deviation (SD).

## Results

The in vitro catalytic efficiencies of various BdPTE mutants (Table 1) towards a broad spectrum of OP substrates (Table 2) were investigated using the well-established AChE inhibition assay (Goldsmith et al. 2016; Job et al. 2020). To this end, all enzymes were produced in the cytoplasm of *E. coli* and purified as functional dimers from the total cell lysate via *Strep*-tag II affinity chromatography as well as ion exchange chromatography as appropriate. Of note, in contrast with earlier studies (Goldsmith et al. 2016, 2017; Cherny et al. 2013), our constructs did not contain the maltose-binding protein (MBP, N-terminal) as fusion partner. The catalytic activities of the variants BdPTE-1 to BdPTE-7 are summarized in Tables 3, 4 and 5.

**Table 2:**
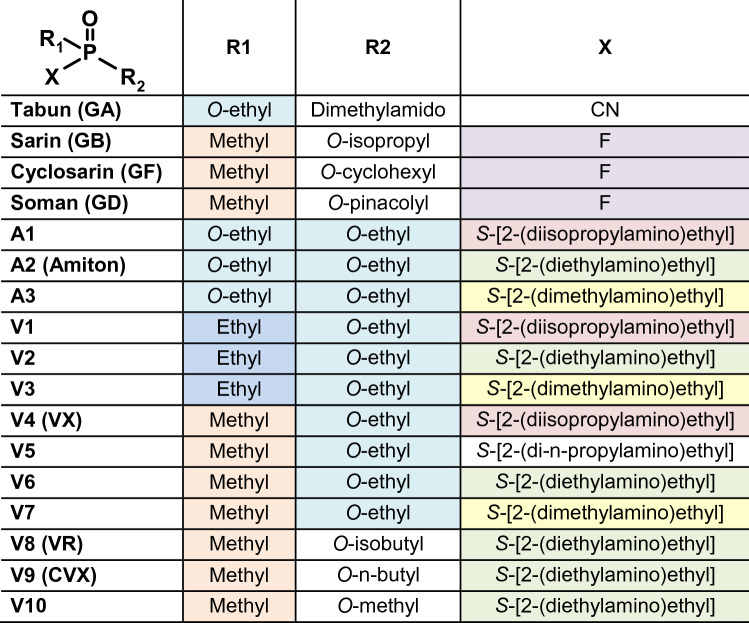
Chemical structures of OPs investigated in this study

**Table 3:** Catalytic efficiencies of BdPTE mutants towards G-type nerve agents

*k*_cat_/*K*_M_ (× 10^6^ M^−1^ min^−1^)	BdPTE-1	BdPTE-2^b^	BdPTE-3	BdPTE-4	BdPTE-5^b^	BdPTE-6	BdPTE-7
GA	225 ± 11.6	647 ± 11.6	582 ± 8.29	313 ± 3.99	136 ± 0.10	133 ± 3.19	380 ± 6.74
GB	106 ± 0.48	165 ± 2.70^a^	200 ± 3.68^a^	142 ± 1.93	52.0 ± 0.82	71.1 ± 1.28	205 ± 11.8
GF	2.14 ± 0.05	5.31 ± 0.21^a^	6.37 ± 0.21^a^	5.03 ± 0.04	10.2 ± 1.49	9.72 ± 0.31	13.6 ± 0.23
GD	4.51 ± 0.08	5.06 ± 0.29	6.80 ± 0.11	4.87 ± 0.08	1.99 ± 0.01	3.23 ± 0.14	7.18 ± 0.05

^a^Data from Job et al. (2020)

^b^Data from Escher et al. (2020)

**Table 4:** Catalytic efficiencies of BdPTE mutants towards V-type nerve agents

*k*_cat_/*K*_M_ (× 10^6^ M^−1^ min^−1^)	BdPTE-1	BdPTE-2^b^	BdPTE-3	BdPTE-4	BdPTE-5^b^	BdPTE-6	BdPTE-7
V1	26.40 ± 0.24	146 ± 4.01	69.4 ± 1.56	49.9 ± 1.56	26.90 ± 0.45	17.20 ± 0.20	38.10 ± 0.36
V2	14.9 ± 0.39	87.6 ± 3.68	38.2 ± 0.51	23.6 ± 0.26	14.50 ± 0.12	9.94 ± 0.16	12.80 ± 0.11
V3	4.21 ± 0.17	16.1 ± 0.03	5.26 ± 0.50	3.05 ± 0.03	3.12 ± 0.09	2.17 ± 0.08	4.33 ± 0.14
V4 (VX)	11.30 ± 0.35	45.9 ± 0.76^a^	63.1 ± 0.24^a^	38.1 ± 0.36	11.80 ± 0.17	16.80 ± 0.47	20.70 ± 0.48
V5	4.41 ± 0.06	10.2 ± 0.03	5.43 ± 0.18	5.21 ± 0.11	1.71 ± 0.02	1.57 ± 0.07	3.45 ± 0.02
V6	5.61 ± 0.43	28.3 ± 0.34	21.8 ± 0.84	16.1 ± 0.04	4.74 ± 0.55	4.24 ± 0.05	9.62 ± 0.39
V7	2.74 ± 0.10	5.69 ± 0.03	2.73 ± 0.13	3.58 ± 0.03	1.18 ± 0.02	1.44 ± 0.04	4.03 ± 0.11
V8 (VR)	1.03 ± 0.004	5.68 ± 0.07	6.06 ± 0.05^a^	5.89 ± 0.26	21.40 ± 0.14	26.20 ± 0.98	22.20 ± 0.66
V9 (CVX)	2.00 ± 0.06	5.98 ± 0.19	6.49 ± 0.22	5.24 ± 0.24	10.60 ± 0.22	11.10 ± 0.34	9.60 ± 0.22
V10	0.30 ± 0.01	0.64 ± 0.01	0.66 ± 0.06	0.56 ± 0.01	2.01 ± 0.003	0.33 ± 0.006	0.90 ± 0.03

^a^Data from Job et al. (2020)

^b^Data from Escher et al. (2020)

**Table 5:** Catalytic efficiencies of BdPTE mutants towards amiton analogues

*k*_cat_/*K*_M_ (× 10^6^ M^−1^ min^−1^)	BdPTE-1	BdPTE-2^a^	BdPTE-3	BdPTE-4	BdPTE-5^a^	BdPTE-6	BdPTE-7
A1	0.25 ± 0.005	0.97 ± 0.03	0.44 ± 0.02	0.66 ± 0.02	0.12 ± 0.002	0.15 ± 0.005	0.22 ± 0.01
A2 (amiton)	0.05 ± 0.001	0.22 ± 0.01	0.14 ± 0.02	0.20 ± 0.005	0.04 ± 0.001	0.03 ± 0.0004	0.08 ± 0.002
A3	0.02 ± 0.001	0.26 ± 0.004	0.02 ± 0.001	0.06 ± 0.003	0.01 ± 0.0003	0.02 ± 0.0004	0.05 ± 0.0004

^a^Data from Escher et al. (2020)

In comparison to BdPTE-1, the further improved variant BdPTE-2 exhibited the highest activities towards GA, GB, GD, most of the V-type nerve agents [V1, V2, V3, VX (V4), V5, V6, V7] as well as several amiton derivatives, whereas the mutant BdPTE-5, with a single amino acid exchange, revealed the highest catalytic efficiencies towards GF, VR (V8), CVX (V9) and V10.

The corresponding variants BdPTE-3 and BdPTE-6, respectively, which were stabilized by eliminating the free thiol side chains, generally showed comparable or even slightly enhanced catalytic activities. Exceptions were the ≥ 2-fold diminished hydrolysis rates of BdPTE-3 for nerve agents with substituents carrying an ethyl and/or O-ethyl group on R1 or rather R2, such as the amiton analogues or the V-agents V1, V2 and V3, and the 6-fold slower degradation rate of V10 by BdPTE-6.

BdPTE-4 (molecular weight: 54,075.93 Da), the C-terminally PASylated version of BdPTE-3, degraded the set of V-type nerve agents with *k*_cat_/*K*_M_ values between 0.56 and 49.9 × 10^6^ M^−1^ min^−1^, G-type nerve agents between 4.87 and 313 × 10^6^ M^−1^ min^−1^ and amiton analogues between 0.06 and 0.66 × 10^6^ M^−1^ min^−1^. Thus, in contrast to BdPTE-3, this variant showed comparable or slightly decreased degradation rates across the entire set of OP substrates investigated here.

The heterodimeric variant BdPTE-7 (molecular weight: 98,502.26 Da), with the subunits of the mutants BdPTE-1 and BdPTE-6 was designed to combine the active sites of each subunit by intramolecular association, and a C-terminal PAS(200)-tag was appended to potentially increase clearance time after in vivo application. Observed *k*_cat_/*K*_M_ values of BdPTE-7 were between 7.18 and 380 × 10^6^ M^−1^ min^−1^ for G-type and between 0.90 and 38.10 × 10^6^ M^−1^ min^−1^ for V-type nerve agents as well as between 0.05 and 0.22 × 10^6^ M^−1^ min^−1^ for amiton analogues. Hence, the substrate spectrum of the fusion protein was significantly broadened compared with the individual BdPTE-1 and BdPTE-6 mutants. For example, 10 nerve agents were hydrolyzed with *k*_cat_/*K*_M_ ≥ 1 × 10^7^ M^−1^ min^−1^ by BdPTE-7, in contrast to only 7 or 8 nerve agents, respectively, by BdPTE-1 or BdPTE-6.

**Fig. 1:**
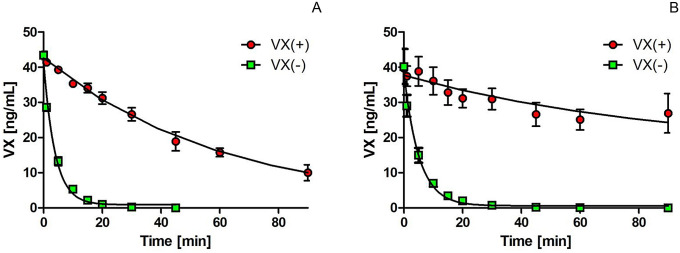
Detoxification of VX by BdPTE-4 (**A**) and BdPTE-7 (**B**): degradation of VX enantiomers was investigated by chiral LC–MS/MS. Red circles VX(+), green squares VX(−). Data are shown as means ± SD (*n* = 3) (color figure online)

Finally, the stereoselective hydrolysis of racemic VX by the most promising variants BdPTE-7 and BdPTE-4 was analyzed using chiral LC–MS/MS (Fig. 1). These measurements revealed a marked stereopreference towards the more toxic P(−) VX enantiomer. At the applied experimental conditions, i.e., a 1142-fold excess of VX with BdPTE-4 and a 600-fold excess with BdPTE-7, BdPTE-4 completely degraded VX(−) within 90 min (*t*_½_ of 3.6 min) and BdPTE-7 within 45 min (*t*_½_ of 2.8 min). Degradation of VX(+) was markedly slower, resulting in *t*_½_ values of 63 and 42 min, respectively.

## Discussion

The present study has demonstrated the potential of further improvement of previously engineered BdPTE mutants, resulting in variants with high catalytic activity towards a broad spectrum of OP nerve agents and preferential degradation of the toxic P(−) enantiomer.

The BdPTE variants C23, 10-2-C3 and 10-2-C3(I106A), here corresponding to BdPTE-1, BdPTE-2 and BdPTE-5, were previously characterized in the form of fusion proteins with the 42.5 kDa MBP with regard to their hydrolytic activity towards different OP nerve agents (Goldsmith et al. 2016, 2017; Cherny et al. 2013). A comparison of the previously published *k*_cat_/*K*_M_ values with those measured in the present study for the variants BdPTE-1, BdPTE-2, BdPTE-5—devoid of a large fusion partner and only equipped with the nine-residue *Strep*-tag II (Schmidt and Skerra 2007)—revealed differences in catalytic activities. Inter-laboratory variations may be due to multiple factors such as experimental conditions (e.g., assay temperature, concentrations of essential cofactors), protein purity and the use of the N-terminal MBP-tag (Roodveldt and Tawfik 2005).

Generally, a catalytic bioscavenger requires a high *k*_cat_/*K*_M_ for efficient detoxification of OPs in vivo. Ashani et al. (2016) compared different animal studies that investigated OP degrading enzymes to define their required circulatory levels. Accordingly, a minimal *k*_cat_/*K*_M_ value of 50 × 10^6^ M^−1^ min^−1^ at an enzyme dose of 1 mg kg^−1^ was proposed to fully protect the animals from intoxication, whereas smaller protein doses (< 1 mg kg^−1^) or degradation rates (e.g. 25 × 10^6^ M^−1^ min^−1^) prevented mortality but not toxic effects. Notably, the enzyme variants BdPTE-1, BdPTE-2 and BdPTE-5 prepared and investigated in the present study showed a catalytic efficiency ≥ 50 × 10^6^ M^−1^ min^−1^ towards the nerve agents GA, GB, V1, V2 and V4 (VX). However, these bioscavenger candidates need further enhancement to cover a broad spectrum of potential threat agents.

To further improve stability of BdPTEs, the mutants 10–2-C3(C59V/C227V) and 10-2-C3(C59V/I106A/C227V) were constructed. By elimination of the two free thiol groups, with the additional substitutions C59V/C227V, the thermal stability of 10-2-C3(C59V/C227V) was raised by 2 °C in comparison with the parental mutant: *T*_m_ = 65.5 ± 0.3 °C versus *T*_m_ = 63.6 ± 0.3 °C) and, in particular, the enzyme was less susceptible to oxidative damage, which may occur in the strongly oxidative environment of the blood stream (Job et al. 2020). Remarkably, the corresponding mutants BdPTE-3 and BdPTE-6 exhibited almost the same catalytic activities as their precursors (BdPTE-2 and BdPTE-5, respectively); however, still only a few nerve agents were efficiently degraded with the desired *k*_cat_/*K*_M_ value of ≥ 50 × 10^6^ M^−1^ min^−1^ (Tables 3, 4).

Efficient detoxification of persistent V-type nerve agents requires not only high catalytic efficiencies but also a long biological half-life (Goldsmith et al. 2017). Methods to prolong circulation of enzymes in vivo, such as chemical coupling with the synthetic polymer poly-ethylene glycol (PEGylation), were previously applied to a BdPTE mutant, thus achieving a mean residence time of 50 h instead of 1 h for the unmodified enzyme (Novikov et al. 2010). An innovative approach to extend the plasma half-life of pharmacologically active proteins including enzymes is PASylation technology, which shares with PEGylation the biophysical effect of retarded glomerular filtration (Schlapschy et al. 2013). However, PASylation offers several advantages over PEGylation (Gebauer and Skerra 2018; Schlapschy et al. 2013): in particular, the ease of manufacturing the PASylated compound as a fusion protein, thus obviating the need for chemical coupling, and the intracellular degradability of the PAS polypeptide, in contrast to PEG, which over time accumulates in cells and tissues. To take benefit of this technology, a PAS tag with 200 residues was appended to BdPTE-3, yielding BdPTE-4, to potentially increase its circulation time in vivo. Moreover, introduction of the flexible PAS sequence as a linker proved useful in the recent investigation of Escher et al. (2020) with the aim to generate a bispecific catalytic bioscavenger. The resulting heterodimer BdPTE-7 combines the substrate profiles of its constituent subunits, BdPTE-1 and BdPTE-6, without impairing their individual catalytic activities. Apart from the PAS(100) linker between the two subunits, the additionally appended PAS(200)-tag should further slowdown clearance in vivo. Although BdPTE-7 did not quite reach the desired *k*_cat_/*K*_M_ values, its extended substrate spectrum, with *k*_cat_/*K*_M_ ≥ 10^7^ M^−1^ min^−1^ for several G- and V-type nerve agents, and its anticipated prolonged circulation make this enzyme a promising drug candidate to protect against OP nerve agent intoxications.

The investigation of the catalytic activities of the PASylated BdPTE-4 and the PASylated fusion protein BdPTE-7 towards VX resulted in *k*_cat_/*K*_M_ values of 38.1 × 10^6^ M^−1^ min^−1^ and 20.7 × 10^6^ M^−1^ min^−1^, respectively, both variants with markedly higher stereochemical preference towards the more toxic VX(−) enantiomer. The knowledge of the substrate-specific *k*_cat_/*K*_M_ values, together with an estimation of BdPTE concentrations after administration in vivo, enables the calculation of OP detoxification half-times using the equation *t*_1__/2_ = 0.69/(*k*_cat_/*K*_M_ × [BdPTE]), an approach which was previously validated with guinea pig in vivo data (Worek et al. 2014). Intravenous injection of 1 mg kg^−1^ BdPTE-4 or BdPTE-7 into a 400 g guinea pig, having 39 mL kg^−1^ plasma volume (Wille et al. 2016), would result in an estimated peak BdPTE plasma concentration of 474 and 260 nM, respectively. On the base of determined *k*_cat_/*K*_M_ values (Table 4), a VX degradation *t*_1/2_ of 2.3 and 7.7 s can be calculated for BdPTE-4 and BdPTE-7. Hence, at the selected BdPTE dose, BdPTE-4 would meet the postulated requirement of a degradation half-time of < 5 s being necessary to preserve survival and to prevent signs of poisoning while BdPTE-7 is close to this requirement.

In conclusion, BdPTE-4 and BdPTE-7 exhibited comparable or slightly enhanced catalytic activities in comparison to their precursor mutants. Further research should be directed towards optimization of catalytic efficiency against a broader spectrum of structurally diverse OP nerve agents. Therefore, the new approach of a heterodimeric scavenger by combination of subunits from different organophosphate hydrolases is considered promising. In fact, the bispecific BdPTE-7 showed a reasonable to high catalytic efficiency towards a broad spectrum of G- and V-type nerve agents and is expected to have a reduced clearance time in vivo due to PASylation. Further in vivo studies are needed to verify the therapeutic efficacy and prolonged circulation times of BdPTE-4 and BdPTE-7 in nerve agent-poisoned animals.
